# Saffron: A Herbal Medicine of Third Millennium

**DOI:** 10.17795/jjnpp-16700

**Published:** 2014-02-20

**Authors:** Hossein Hosseinzadeh

**Affiliations:** 1 Pharmaceutical Research Center, Department of Pharmacodynamy and Toxicology, School of Pharmacy, Mashhad University of Medical Sciences, Mashhad, IR Iran

**Keywords:** Crocus, Herbal Medicine, Molecular Mechanisms of Pharmacological Action

Saffron, the dried stigma of the plant *Crocus sativus* L. (a member of Iridaceae family) has a distinct color, flavor and smell. It is widely used as a spice, and as a coloring and flavoring agent in the preparation of foods and cosmetics. According to chemical analysis, more than 150 chemicals are present in saffron stigmas among which, the three main chemical compounds including crocins (mono and diglycosyl esters of a polyene dicarboxylic acid, named crocetin), picrocrocin (a precursor of safranal), and safranal (monoterpen aldehyde) are responsible for saffron exclusive color, taste, and odor, respectively (1-3). Various pharmacological activities of saffron and its constituents have been extensively studied including: anticancer, antidepressant, anti-Parkinson, anti-Alzheimer, anticonvulsant, anti-ischemic (such as brain, kidney, muscular and heart ischemia), anti-hypertensive, anti-genotoxic, and antidote (e.g. against snake venom, diazinon, acrylamide or acrolein), antitussive, hypolipidemic, antioxidant, antinociceptive, and anti-inflammatory effects. Some clinical studies about saffron and its constituents have been cited in the literature such as safety evaluation, aphrodisiac, antidepressant, and anti-Alzheimer effects (3-5).

In traditional medicine in various countries, saffron has been used for various purposes including analgesic and anti-inflammatory (earache, tooth-ache, swelling, otitis, anal pain, gout, cancer pain, gingivitis, discomfort of teething infants), cardiovascular system (cardiac stimulant, removes blockages of vascular), central nervous system (narcotic, antihysteric, CNS stimulant, hypnotic, mental disease, sedative, anticonvulsant, neurasthenia), eye disease (painful eye, lacrimation, day blindness, corneal disease and cataract, purulent eye infection, pterygium, poor vision), gastrointestinal system (stomachic, anorexia, treatment of hemorrhoid, prolapse of anus, jaundice, and enlargement of the liver, antiflatulent), genitourinary system (abortion, treatment of amenorrhea, aphrodisiac, impotency, emmenagogue, stimulate menstruation, prolapse of anus, stop menstrual periods, promote menstruation, use in puerperium period, terminate pregnancy, painful urination, diuretic, kidney stone), infection disease (antibacterial, antiseptic, anti‐fungal, measles, smallpox, scarlet fever), respiratory system (asthma, bronchitis, expectorant, pertussis, dyspnea, pleurisy, antitussive, diphtheria, disability tonsils resulting snoring, respiratory decongestant, expectorant), skin disease (treatment of psoriasis, eczema, acne, wound), and miscellaneous (immunostimulant, diaphoretic, tissue coloration, anticancer) (6). Saffron and its constituents have shown multiple useful effects, especially on CNS and against cancer. However, clinical evidence is still scarce in this regard and more comprehensive studies with special focus on human clinical trials is required.
